# TRanslational Initiative to DE‐risk NeuroTherapeutics (TRIDENT) – A revolutionary pre‐clinical approach to maximize the predictive validity of pre‐clinical evaluation

**DOI:** 10.1002/alz70856_104027

**Published:** 2025-12-25

**Authors:** Sriram Jayabal, Ali R. Khan, Edward A Fon, Jaqueline Sullivan, Joel Watts, Justine Clery, Liisa AM Galea, Lisa M Saksida, Mallar M. Chakravarty, Marco AM Prado, Richard Gold, Thomas M. Durcan, Timothy J Bussey, Vania F Prado, Ravi S. Menon

**Affiliations:** ^1^ Western University, London, ON, Canada; ^2^ Montreal Neurological Institute, McGill University, Montreal, QC, Canada; ^3^ University of Toronto, Toronto, ON, Canada; ^4^ McGill University, Montreal, QC, Canada; ^5^ Centre for Addiction and Mental Health, Campbell Family Mental Health Research Institute, Toronto, ON, Canada

## Abstract

**Background:**

Neurodegenerative diseases such as Alzheimer's Disease (AD), Frontotemporal Dementia (FTD), Dementia with Lewy Bodies (DLB), Parkinson's Disease (PD), and Multiple System Atrophy (MSA), are complex and poorly understood conditions characterized by progressive neuronal degeneration, affecting brain structure, function, and behavior. Cognitive impairment is seen in all these disorders despite heterogeneous clinical presentation, even in primarily motor diseases such as PD, causing hardship for the individuals and their caregivers. Dementia alone presents a growing public health crisis globally. Canada's annual dementia‐related healthcare costs are projected to rise from $19.7 billion in 2011 to $92.8 billion by 2031. Additionally, the lack of effective treatments and the high failure rate (92%) of clinical trials for new central nervous system (CNS) therapies underscores the immediate need for effective pre‐clinical approaches that can reliably predict success in clinical trials. High failure stems from four key issues: neglect of human‐relevant cognitive measures, lack of sex and age factors, reliance on single‐investigator studies, and poor reproducibility due to limited collaboration and sociocultural barriers.

**Method:**

The **TRanslational Initiative to DE‐risk NeuroTherapeutics (TRIDENT)**, with its novel platform for pre‐clinical evaluation, addresses these critical gaps. TRIDENT integrates three advanced model systems— newly developed human‐induced pluripotent stem cells (iPSCs) and organoids, as well as new humanized mouse and marmoset models—into a unified platform to dramatically improve the predictive validity of the pre‐clinical to clinical outcomes. TRIDENT leverages the human‐relevant touchscreen‐based cognitive tasks in mice and marmosets, which are highly translatable to humans. Further, TRIDENT incorporates Sex‐Based Analysis (SBA)+ and open science principles across all stages of preclinical evaluation, fostering inclusivity, collaboration, reproducibility, and accessibility. With testing facilities at McGill University, the University of Toronto, and the Center for Addiction and Mental Health, TRIDENT ensures robust multi‐site validation of results. Currently, TRIDENT is evaluating compounds for alpha‐synucleinopathies and AD using these models to validate the platform.

**Result:**

TRIDENT offers a revolutionary solution for developing effective therapies by maximizing predictive validity and de‐risking the drug discovery process.

**Conclusion:**

This will transform the current approaches of pre‐clinical testing in neurodegenerative disorders, making the process attractive to pharmaceutical and biotechnology industries to find effective cures and therapies.

